# Mass Contributions in London

**Published:** 1922-12

**Authors:** E. W. Morris

**Affiliations:** C.B.E., House Governor, London Hospital


### MASS CONTRIBUTIONS IN LONDON.

### THE HOSPITALS SAVINGS ASSOCIATION SCHEME.

By E. W. MORRIS, C.B.E., House Governor, London Hospital.

HITHERTO, in the long history of the voluntary hospitals, it has been acknowledged that a voluntary hospital is a charity, and that it deals with two classes of the public, namely, those who support the charity and those who benefit by the charity—the givers and the receivers ; and these two classes have been kept apart, and distinct. It has always been recognised that the giver gives, not for his own good, but for someone else's, and that the receiver receives because someone else has paid the cost. Many things have happened in more recent years which have tended to obliterate this sharp line of distinction between donor and recipient, and we seem to be approaching the time when the giver and the receiver may be one and the same person.

The principal cause for this coming change is the increasing complexity of medical science, and this, in turn, has made medical diagnosis and medical treatment more costly. How costly it is becoming is only too well known to all of us older hospital men. We look back almost with envy, unworthy envy, to those happy days when a hospital bed cost less than £2 a week! Then came, one after the other, great discoveries which were at once applied to curative processes, and we opened Röntgen Ray departments, Finsen Light, bacteriological, chemical, electrical, inoculation, massage and other departments. The honorary staff became more and more specialised, and we had to meet the expensive equipments of ophthalmic, aural, dermatological and gynæcological departments. And our "cost per bed" crept up from £2 to £3, then £4, then £5, and is now on the way to £6 per week. The recurring successes over this or that form of disease, the falling death rate, the more perfect recovery—filled us with delight. But the cost! That fills us with despair.

This increasing cost of diagnosis and subsequent treatment is the principal cause why the donor and the recipient are becoming one and the same person. My old minutes speak of "generous donors" and "miserable objects." But now, the generous donor of one year may be the miserable object of next, for the giver—the small giver, at any rate—while quite willing to give for the general good, feels that he himself should be able to benefit in the general good should occasion arise. He simply cannot afford the cost of complete and methodical examination such as a hospital patient secures, just as the destitute could not afford the simple treatment of long ago.

### The Real Tragedy.

But while the increased cost is the cause of patients asking the hospital's help—patients of a class and social standing for which the hospital was not originally intended—this increased and increasing cost has another effect ; it is ruining the hospitals, bankrupting them. It should be properly appreciated that the tragedy of a starving hospital is far greater than can be measured by closed beds, though if you have stood in the receiving-room of a busy hospita and have seen the pathetic anguish of mother or husband when child or wife is turned away for no other reason than that there is "no bed," you will think that is tragedy enough. The tragedy of tragedies is that the hospital ceases to be a fighter ; laboratories are starved of equipment and staff ; new discoveries cannot be used ; improved methods cannot be tried. And when a hospital ceases to be a fighter, then the great army of disease which was being pushed back presses forward again. I am assuming that it is agreed that the value of a hospital's work extends far outside its walls—in the doctors and nurses it trains ; in the information it spreads ; in the gospel it preaches.

And so this increased costliness of medical diagnosis becomes the direct cause of two effects :—

(1) That the man of average means, in receipt of regular wages or small income—far from destitute—wants to come into hospital when he is ill because he cannot possibly afford such investigation and subsequent treatment in private. But he would like to contribute towards the cost.

(2) The hospitals are bankrupt because they cannot afford the outpouring of money which modern research demands.

These two effects mutually react on each other. The man wants to pay. The hospital badly wants the money.

The result could easily be foreseen, and most hospitals now accept part payment. The London Hospital, for more than a year, has charged a guinea a week (one-fifth of the cost) to all patients, except children, and has increased its income by £20,000 by this means.

### Honourable Liars.

But the finding of a guinea a week by the patient, who may be the wage-earner, when he is himself ill, is difficult and often impossible. Every care is used when arranging with a patient for this guinea that no hardship be done, and if everyone told the truth our work would be easy. But while there are dishonourable liars, there are honourable liars, too, who, on admission, declare they can well afford the guinea, and of whom we discover afterwards that they could not. A month ago we admitted a fisherman of this breed from the South Coast ; he told us he could well afford a guinea ; his illness progressively got worse, and at last he became blind and his letters had to be read to him. One of these was from his wife and the whole truth came out—the magnificent pluck ; the sick baby ; the small sums borrowed from neighbours—all made so light of—"it is nothing—we are all right—it is worth it, if they can but get you well, dear. You will come back and dress baby's tree at Christmas, and we shall all be happy again. Baby is not quite well to-day ; he will be better to-morrow." Poor fellow, he will never see baby's Christmas-tree. But the letter made me hate the guinea charge; it may be so cruel. And do we not all know how recovery is delayed, even prevented, because the patient is in constant mental unrest as to what is happening at home?

THE PROVINCIAL EXAMPLES.

It is because most of us feel that, while generally right and fair, it may be cruel, that we have looked round for some other means of benefiting the hospitals without risk of distress to the deserving. Some arrangement whereby by paying small sums when well, free treatment could be secured when ill. Some such scheme was in force in the provinces, and we sent one of our officers to the following cities and towns to examine their schemes on the spot: Glasgow, Edinburgh, Newcastle, Sunderland, Leeds, Sheffield, Derby, Leicester, Norwich and Oxford. It would occupy much too much space to give his report of this most instructive tour, but the common factors may perhaps be summarised.

At all these towns a serious effort is made by the workmen population to save the voluntary principle, which they prefer. Regular weekly contributions are made to the hospitals either directly or through a hospital society by the great workshops, warehouses, factories, works and collieries. The contributions are often but not always deducted from the wages "at source." There are various ways of fixing the rate of contribution to the hospital. Sometimes a flat rate of, say, 2d., 3d., or 4d. a week from each workman; sometimes a proportion of wages earned (*e.g.*, 1d. per 20s. of wages earned).

Often the owners or directors contribute, too, their contribution having a relation to the amount paid by the men—*e.g.*, sometimes one-third is added to the collection from the men; or the directors vote 2s. per annum to the hospital for each man in their works; or their contribution has a relation to the weekly wage paid (£1 per £1,000).

WHY THERE IS NO CONTRACT.

In all cases free treatment is given to members at the hospitals, both as out and in-patients. There is no contract to admit, because it is not possible under present conditions to increase beds, and the hospital must retain its right to say what is a hospital case, and the men understand this; the agreement is that if and when admitted there shall be no charge at all, and in some cases it is agreed there shall be no appeal for further contributions.

When a man requires help from the hospital he usually gets a voucher from the secretary of the hospital fund of his group, and presents it at the hospital. The hospital obtains payment on these vouchers from the hospital association of the town. The various contributing groups are represented on this association, which often provides other benefits than strictly hospital ones, such, for instance, as convalescent home and appliances (trusses, teeth, &c.).

In all cases where mass contributions had been organised, the hospital funds have been largely increased, and, what is most important, a regular income has been assured. In all cases the hospital authorities alone determine whether a case ought to be admitted or not, and are influenced only by the severity of the case.

In some cases representation on the management of the hospital is given to representatives of the men, but usually on the association working the scheme.

THE SOCIAL ELEMENT.

In all cases every endeavour is made to create and to keep up the interest of the men in the hospital's work. It is *their* hospital, and it would be bad form not to help it. Tours of the hospital on Saturday afternoons and evening lantern lectures on phases of hospital work are regularly provided. There is always a definite inducement for members to join—*e.g.*, "Help others and possibly yourself"; "No help, no hospitals"; "Freedom from charges."

Everywhere we were urged to start such a scheme in London and its success was foretold. Fortunately such a scheme is about to start, and the "London" and many other hospitals have decided to co-operate.

THE NEW SCHEME.

A Hospital Savings Association has been formed. On the provisional Executive Council are men whose names are familiar in the hospital world, and outside it:—

Viscount Hambleden (King's College Hospital).
Sir Alan Anderson, K.B.E. (Hon. Secretary of King Edward's Hospital Fund).
Mr. W. A. Appleton, C.B.E.
Mr. Stuart de la Rue.
Viscount Goschen (Guy's).
Viscount Knutsford (London).
Gen. the Hon. Sir H. A. Laurence.
Mr. Henry Lesser.
Sir Edward Penton, K.B.E.
The Hon. Sir Arthur Stanley (St. Thomas's).
Mr. J. F. Stirling (Hospital Saturday Fund).

I cannot do better than quote from a pamphlet drawn up by the Association:—

The Hospital Savings Association has been set up with the co-operation of most of the leading London hospitals to provide a means by which wage-earners may, by saving regularly for the hospitals in their place of employment, club, &c., and by pooling their savings, collectively reimburse the hospitals for services rendered to individuals among them, and be relieved from all payment when they or their families receive hospital treatment.

The hospitals need a larger and more assured income; for want of it very many beds have been closed in the last year or two. The wage-earner may need the hospital any day, and does not want to make heavy payments at the hospital at a time when he can least afford it.

PRIVILEGES OF CONTRIBUTORS.

When a contributor, his wife or children under sixteen has been admitted to treatment by any co-operating hospital he will be exempted—

(*a*) From Almoner's inquiry as to means, and
(*b*) From any payment either as out-patient or in-patient.

In suitable cases assistance will be given to contributors in obtaining surgical appliances, glasses, dental treatment, convalescent treatment, and ambulance services.

CONDITIONS.

(1) Payment of a regular contribution of 12s. per annum in advance or 3d. per week.
The above contribution may be made by employer and employees jointly.

(2) The decision as to whether any case is suitable for hospital treatment and the order of priority in which applicants (whether contributors or not) are admitted, lies with the individual hospital.

(3) A contributor's income must be within hospital income limits, as follows :—

| | |
|---|---|
| Single man or woman ... ... | £4 per week. |
| Married without children under 16... | £5 ,, |
| Married with children under 16 ... | £6 ,, |

(4) No exemption from payment at the hospital is given in ordinary maternity cases, or in any case where provision for treatment is made by the State or Local Authorities.

REPRESENTATION OF CONTRIBUTORS.

The views of contributors will be fully represented on the Association, the membership of which will be divided into three classes—Hospital Members, Contributor Members and General Members. The business of the Association will be conducted by the members through an Executive Council elected annually from among the members, and each class of member will be entitled to elect one-third of the Council.

Further particulars can be obtained from Mr. F. B. Elliot, C.B.E., General Secretary, Hospital Savings Association, 19 Berkeley Street, W.1.

LOCAL PATRIOTISM OF LONDONERS.

Personally I believe that the salvation of our hospitals and of the voluntary system lies in some such scheme as the above, and the "London" intends to do all in its power to help to make it a success. It has succeeded in the Provinces, and should succeed in London. I know that in London one of the drawbacks is the lack of "town patriotism." In the Provinces the hospital is the pet charity of the town, and everyone supports it. In Greater London there are 102 hospitals, and the sense of proprietorship in *the* hospital is lacking. Nevertheless, by the very fact of the number of hospitals, general and special, the value of such an Association is increased ; for vouchers are of equal value wherever presented, and the wage-earner and his family may attend the same or different hospitals at their convenience or choice. And it will be of great assistance to members to be able to communicate with the Association Headquarters to find out where a suitable bed may be vacant, and thus put an end to the present scandal of a patient zig-zagging through London to find one.

Such a scheme will enable the hospitals to take part in the great forward movement in the victory over disease, and the gifts of the charitable can be spent to better purpose than ever. Predisposing causes of our great scourges can be looked into, and their connection with home conditions or occupation conditions, or with heredity, made clear. The time is ripe for great discoveries and the application of these great discoveries to curative and preventive work. Pasteurs, and Jenners, and Listers are still amongst us—men whose genius, given a chance, can make the world a better place to live in. The starvation of the hospitals can give no chance to such men, but I think their time is coming now.

